# Supplementary material for: The UBP5 histone H2A deubiquitinase counteracts PRCs-mediated repression to regulate Arabidopsis development
Source: Nat Commun. 2024 Jan 22;15:667. doi: 10.1038/s41467-023-44546-8 (PMC10803359; doi:10.1038/s41467-023-44546-8)
Supplement: Supplementary file 1 — Supplementary Information [file 41467_2023_44546_MOESM1_ESM.pdf]

The UBP5 histone H2A deubiquitinase counteracts PRCs-mediated repression to regulate Arabidopsis development

Godwin *et al.*

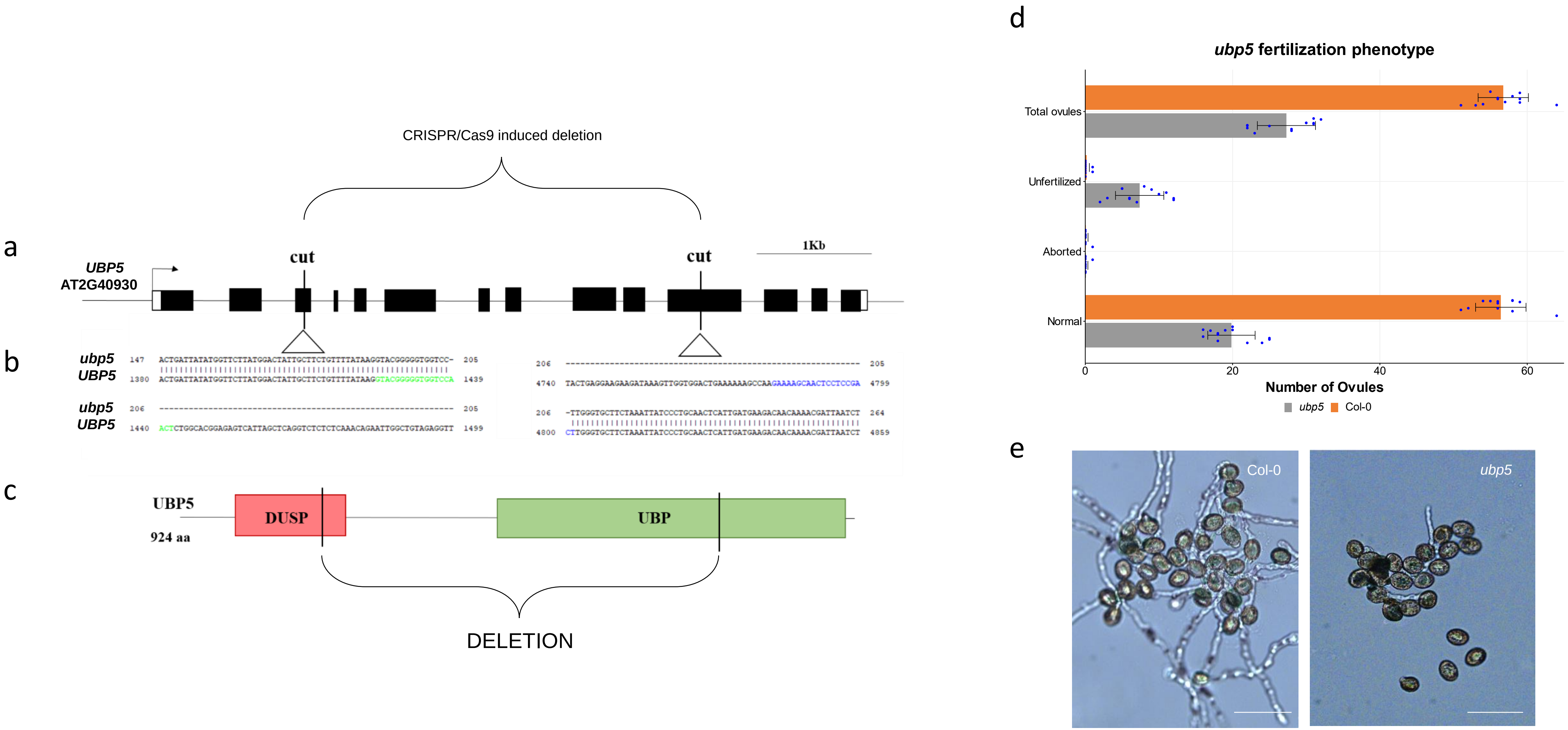

Supplementary Figure 1. *ubp5* is a CRISPR/Cas9 induced deletion mutant with fertilisation defects. a, Schematic representation of the *UBP5* (At2G40930) gene indicating the genomic region that was deleted by CRISPR/Cas9 in *ubp5* (black boxes, exons; white boxes, UTRs). b, Confirmation of the borders of the deletion in *ubp5* by sequencing. sgRNA regions marked respectively in green and blue. c, Schematic representation of the UBP5 protein indicating the deleted region in the protein encoded by the *ubp5* allele. d, Fertilisation analyses of *ubp5*. Fertilised versus normal ovules, aborted and unfertilised ovules in siliques were counted under the microscope (n = 12 siliques were used for Col-0 and *ubp5*). Error bars represent the standard deviation (SD). e, In vitro pollen germination assays showing *ubp5* pollen germination defects. Pollen collected from Col-0 and ubp5 plants. n=3 biological replicates. Scale bar 100 µm.

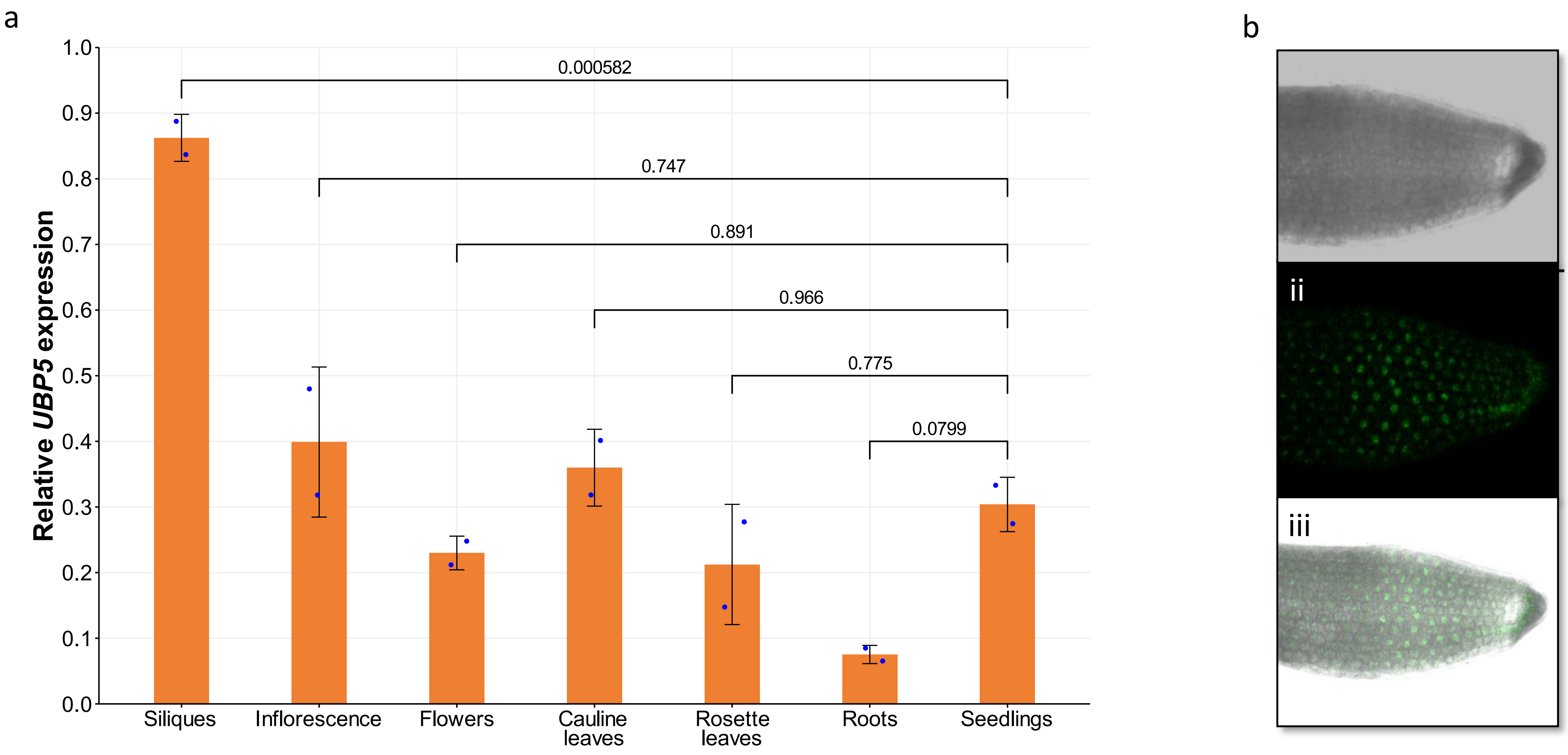

Supplementary Figure 2. *UBP5* expression in different tissues. a, Relative expression analysis of *UBP5* in Col-0 siliques, inflorescences, flowers, cauline leaves, rosette leaves, roots and seedlings. Two biological replicates were used for this experiment. Significant differences were determined by the one-way ANOVA with post hoc Tukey's multiple comparison test. Different letters indicate significant differences of expression at $P < 0.05$ level. b, UBP5-GFP localisation in root nuclei from 5-day-old seedlings. *UBP5pro::gUBP5-GFP;ubp5* was used for this analysis (i, brightfield; ii, GFP channel; iii, overlay). Scale units (10.5 µm).

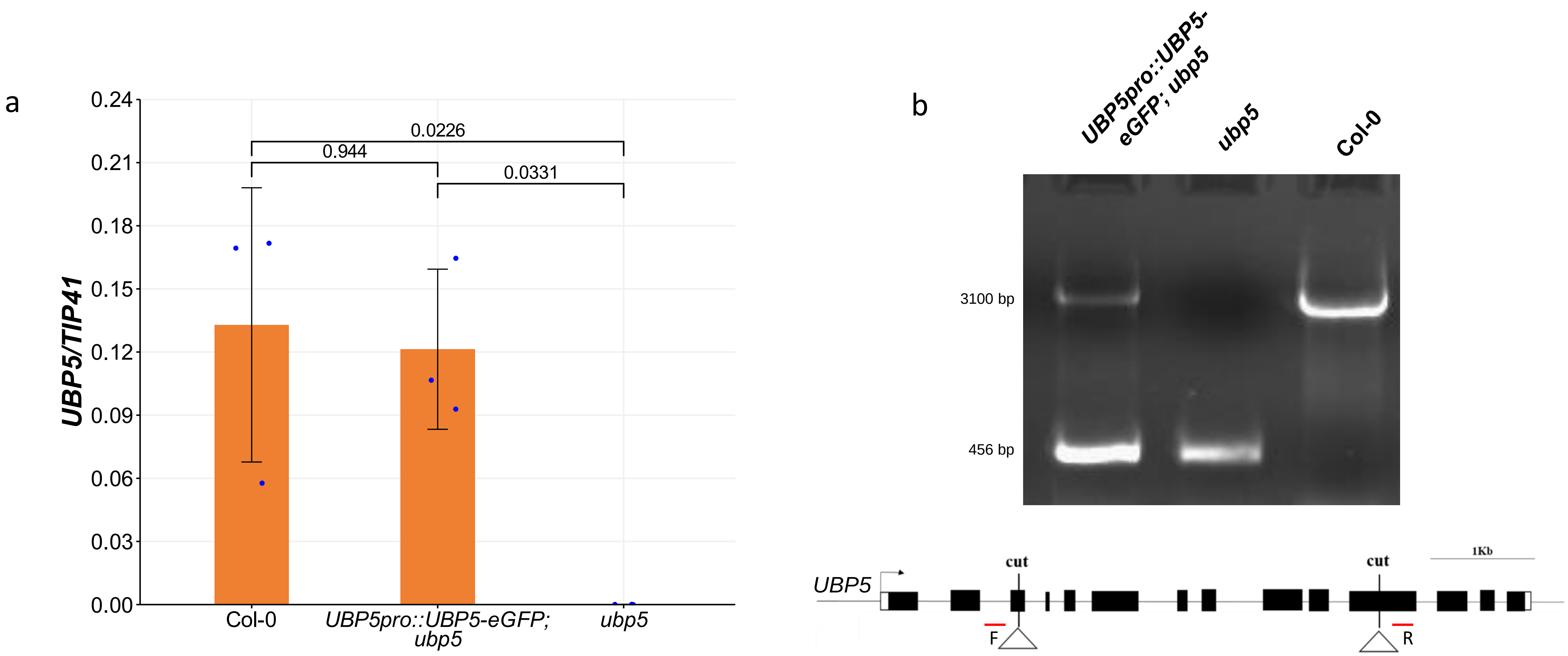

Supplementary Figure 3. Complementation of the *ubp5* mutant by *UBP5pro::gUBP5-eGFP*. a, RT-qPCR analysis of *UBP5* expression in Col-0, *UBP5pro::gUBP5-eGFP;ubp5* and *ubp5*. Error bars indicate standard deviation (SD) of three biological replicates (n=3), grown and harvested independently. Significance tests were done using ANOVA with post hoc Tukey's test and p-values are indicated above plot. b, Genotyping results using forward (F) and reverse (R) primers showing that the complementing line *UBP5pro::gUBP5-eGFP;ubp5* contains both wild type (upper band) and *ubp5* (lower band) alleles.

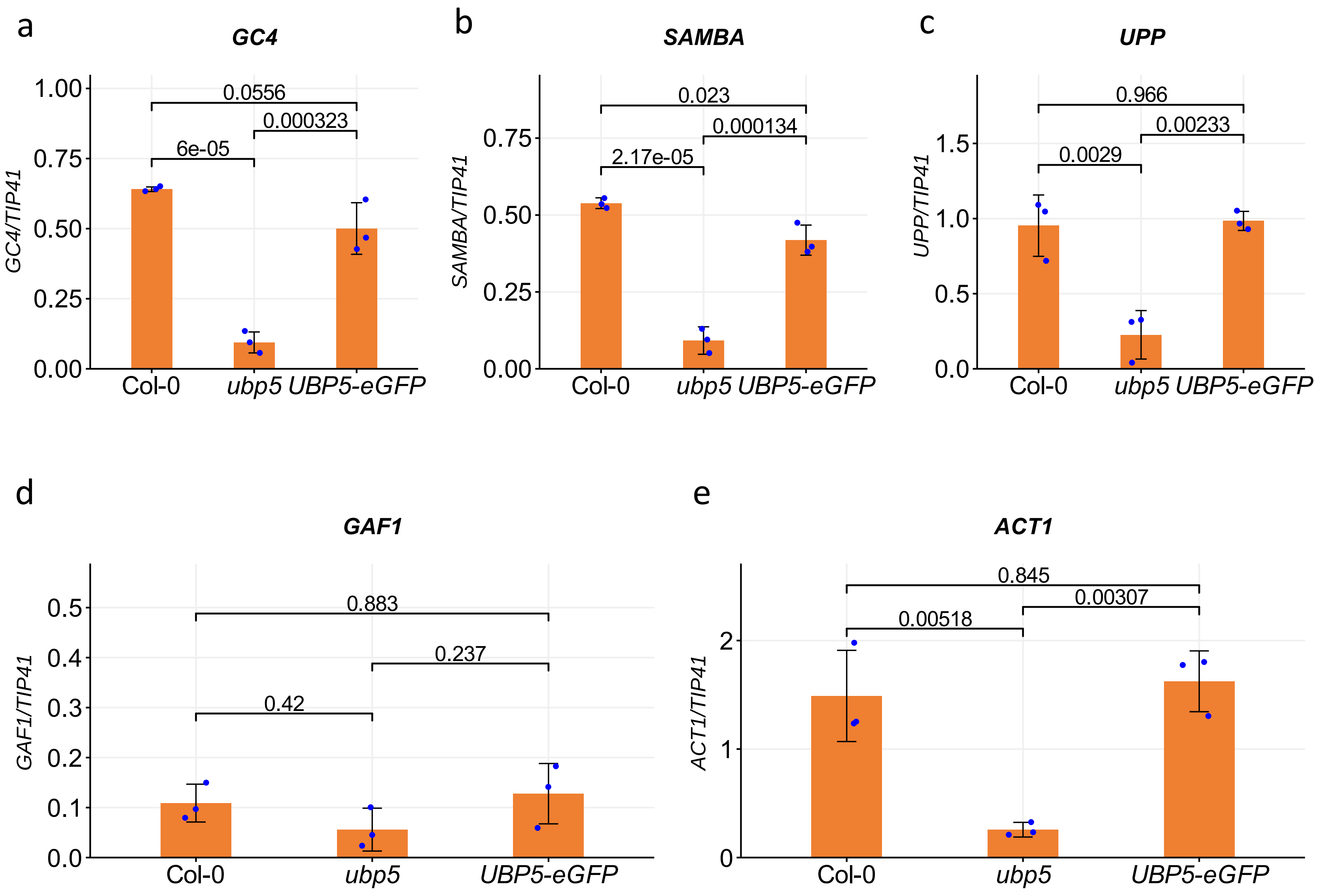

Supplementary Figure 4. Expression analyses of developmental target genes in *ubp5* and *UBP5pro::gUBP5-eGFP;ubp5* (*UBP5-GFP*). a-e, RT-qPCR analyses showing the expression of UBP5 target genes which showed increased H2Aub and H3K27me3 levels in *ubp5*: a, *GOLGI CANDIDATE 4* (*GC4*); b, *SAMBA*; c, *URACIL PHOSPHORIBOSYLTRANSFERASE* (*UPP*); d, *GAMETOPHYTE DEFECTIVE* (*GAF1*); e, *ACTIN 1* (*ACT1*). Error bars indicate SD, N= 3 biological replicates, grown and harvested independently. Significance tests were done using ANOVA with post hoc Tukey's test and p-values are indicated above plot.

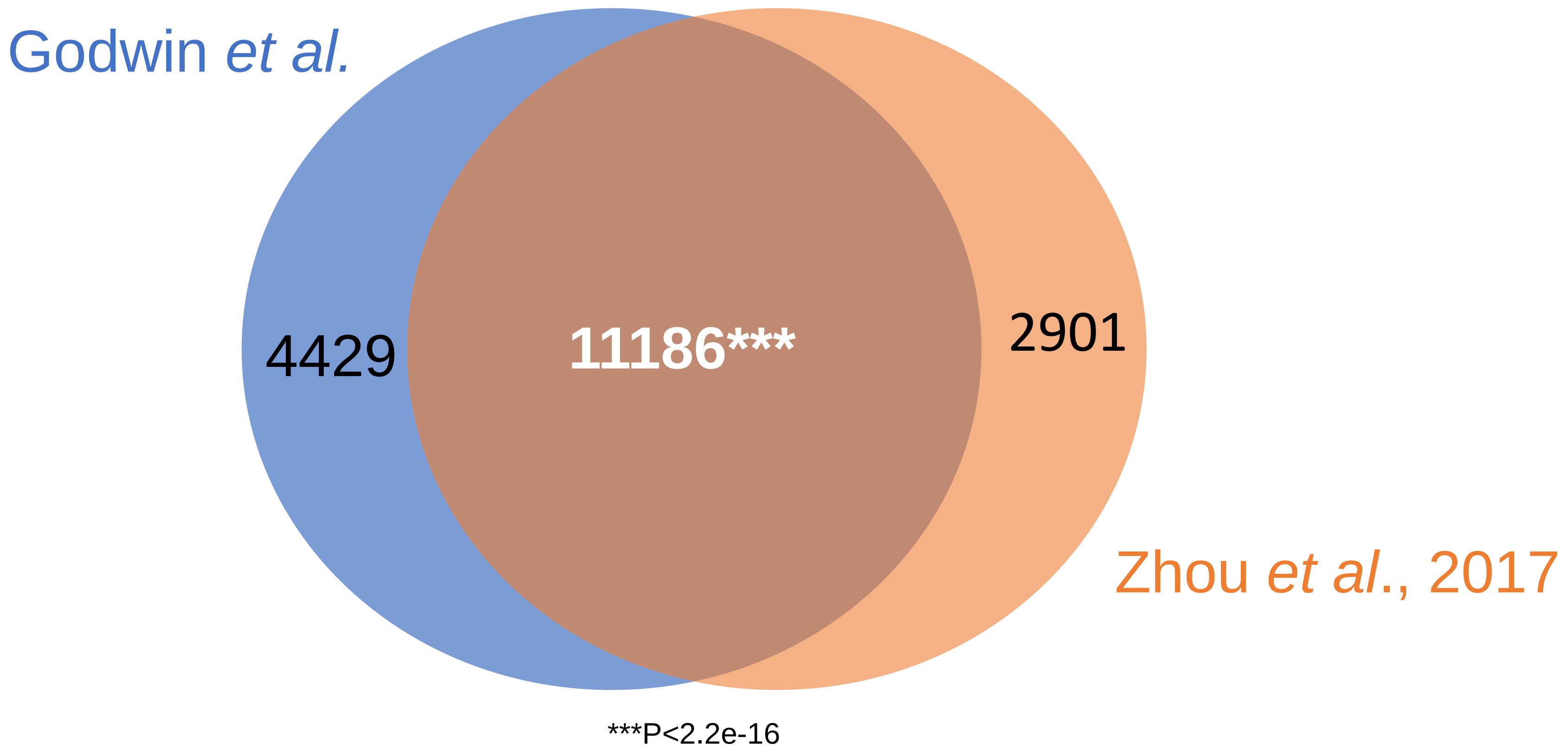

Supplementary Figure 5. H2Aub marked genes between different datasets in Col-0 seedlings. Venn diagram representing the high overlap of H2Aub marked genes in Col-0 between our dataset and Zhou et al., 2017 dataset.

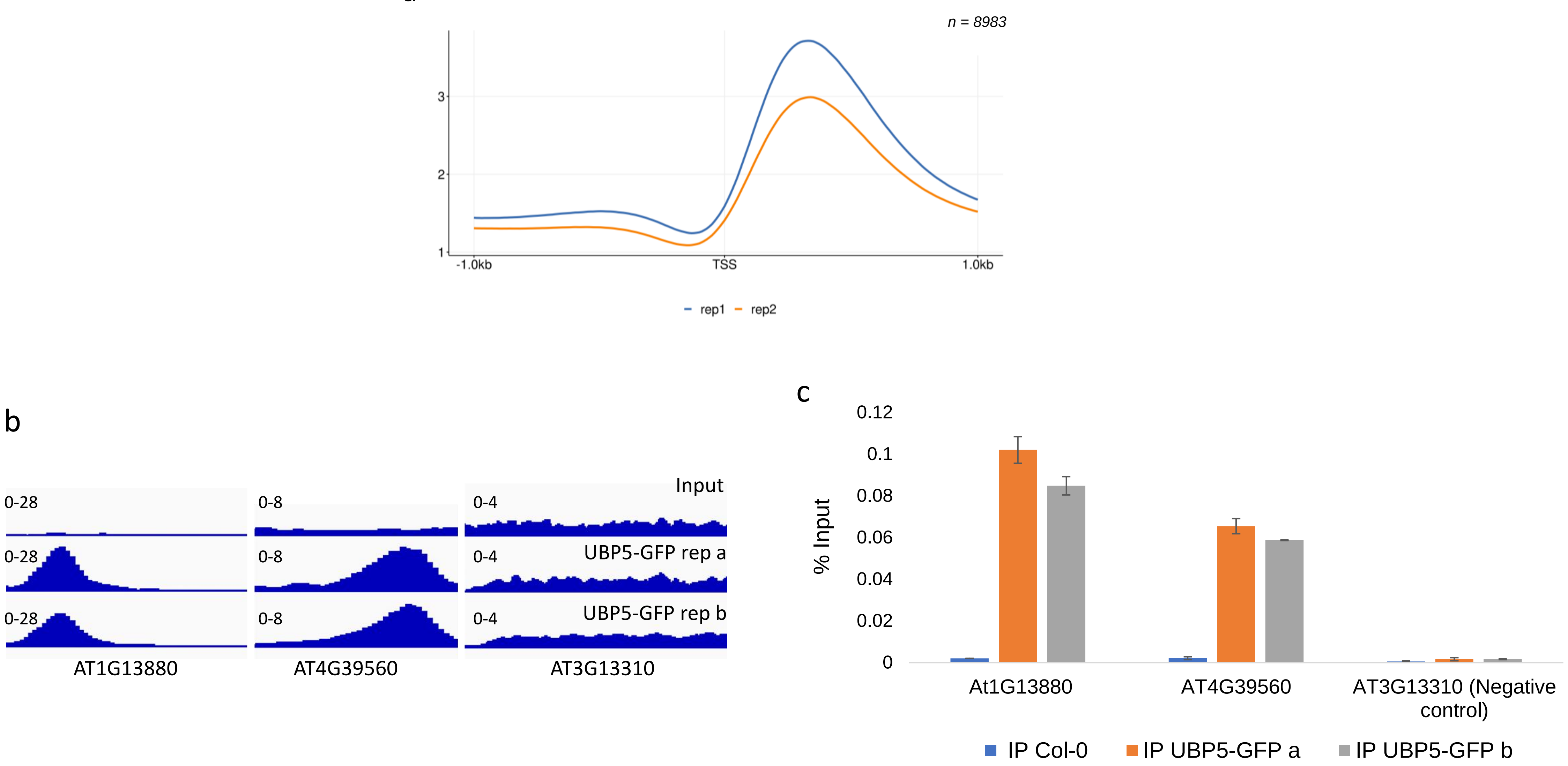

Supplementary Figure 6. UBP5 enrichment at representative loci. a, Metagene plot showing that individual biological replicates of UBP5 ChIP-seq. b, IGV browser view of UBP5 peaks from immunoprecipitated samples of *UBP5pro::gUBP5-eGFP;ubp5* (UBP5-GFP) from two biological independent replicates a and b compared to input. c, ChIP-qPCR results for UBP5 target loci AT1G13880 and AT4G39560 in biological replicates a and b measured as % of input. AT3G13310 was selected as a negative control where no peak was observed in the IGV browser. N=2 biological replicates. Error bars indicate SD.

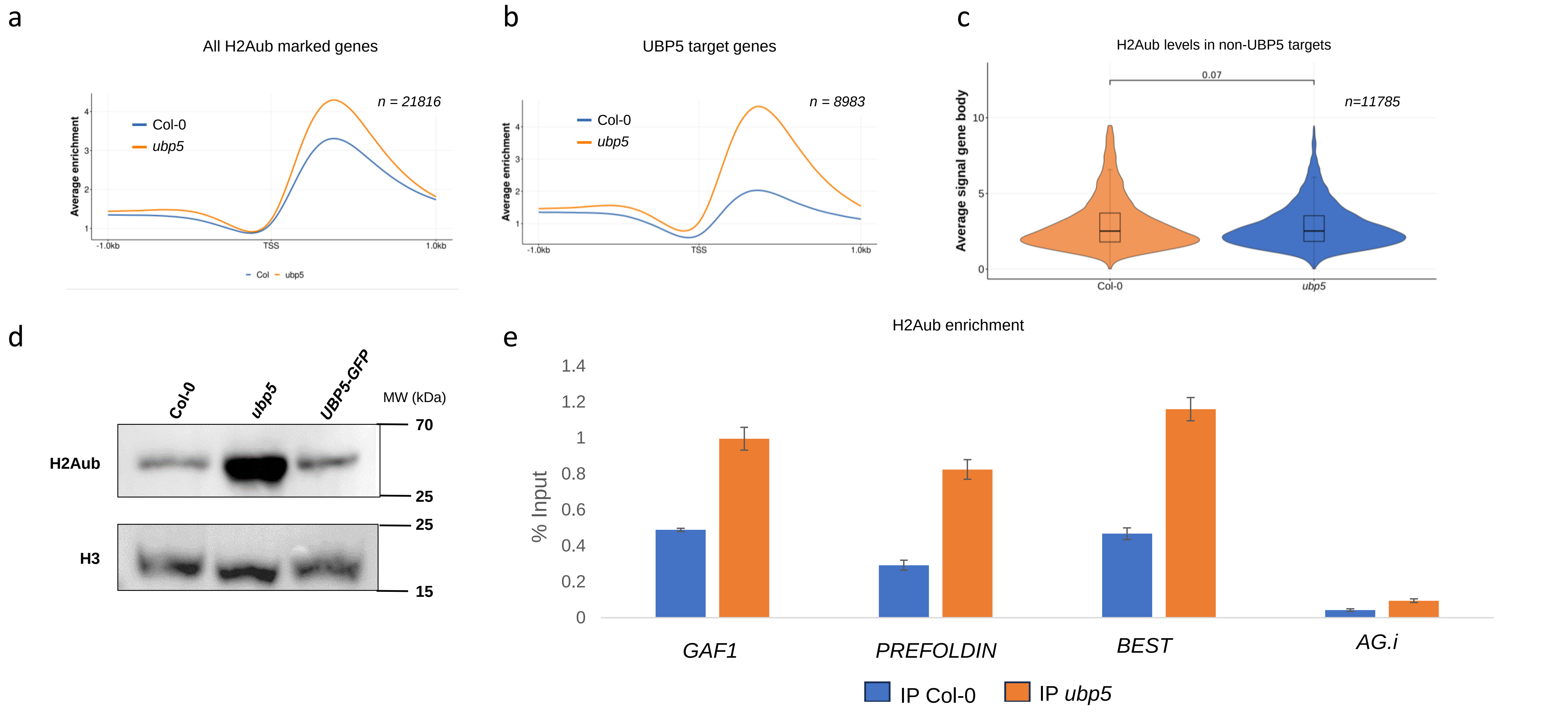

Supplementary Figure 7. UBP5 binding overlaps with the gain on H2Aub in *ubp5*. a-b, Metagene plot of H2Aub distribution in (a) all H2Aub marked genes and (b) UBP5 targets genes. c, Average signal of H2Aub at gene body for non-UBP5 targets in Col-0 and *ubp5*. Statistical significance is tested according to one-sided Wilcoxon rank sum test, p-values are indicated above the plot. The violin plots show the distribution pattern of data and are overlaid with boxplots. For box and whiskers plot the middle line represents the median; the upper and lower lines are the first and third quartile (Q1 and Q3); the whiskers indicate the upper and lower limits of data spread by subtracting 1.5* interquartile range (IQR) from Q1 and adding 1.5* IQR to Q3. d, H2A deubiquitination assay in *N. benthamiana* cells. H2Aub levels from seedlings of Col-0, *ubp5* and *UBP5pro::gUBP5-eGFP.* Histone H3 is used as a loading control. e, ChIP-qPCR validation of H2Aub hyper-marking in ubp5. Selected loci (*GAI ASSOCIATED FACTOR 1* (*GAF1* - AT5G59980), *PREFOLDIN* - AT1G03760 and *BESTROPHIN-LIKE PROTEIN* (*BEST* - AT3G61320)) were among the 207 UBP5 targets downregulated in *ubp5*. An intergenic region (*AG.i*) which is not H2Aub enriched was selected as negative control. ChIP-qPCR results shown as % of input DNA. This data is a representation of one biological replicate. Error bars indicate SD of two technical replicates.

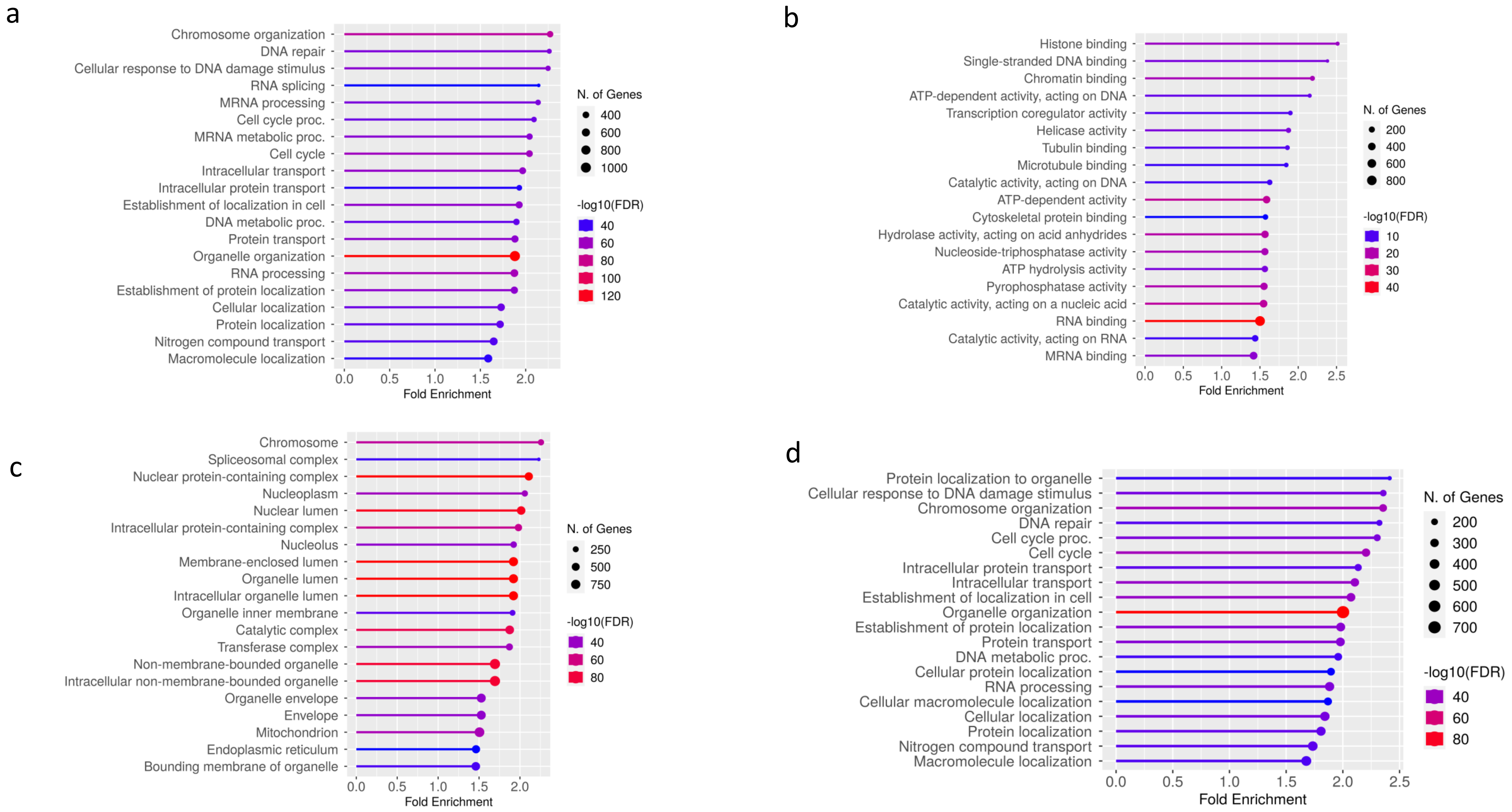

Supplementary Figure 8. Functional categorisation of UBP5 target genes in ShinyGO analysis. a-b, GO analysis of all UBP5 target genes based on a) biological process; b) molecular function; and c) cellular component. d, GO analysis of UBP5 targets which gained H2Aub in ubp5 based on biological process. False Discovery Rate (FDR) < 0.05.

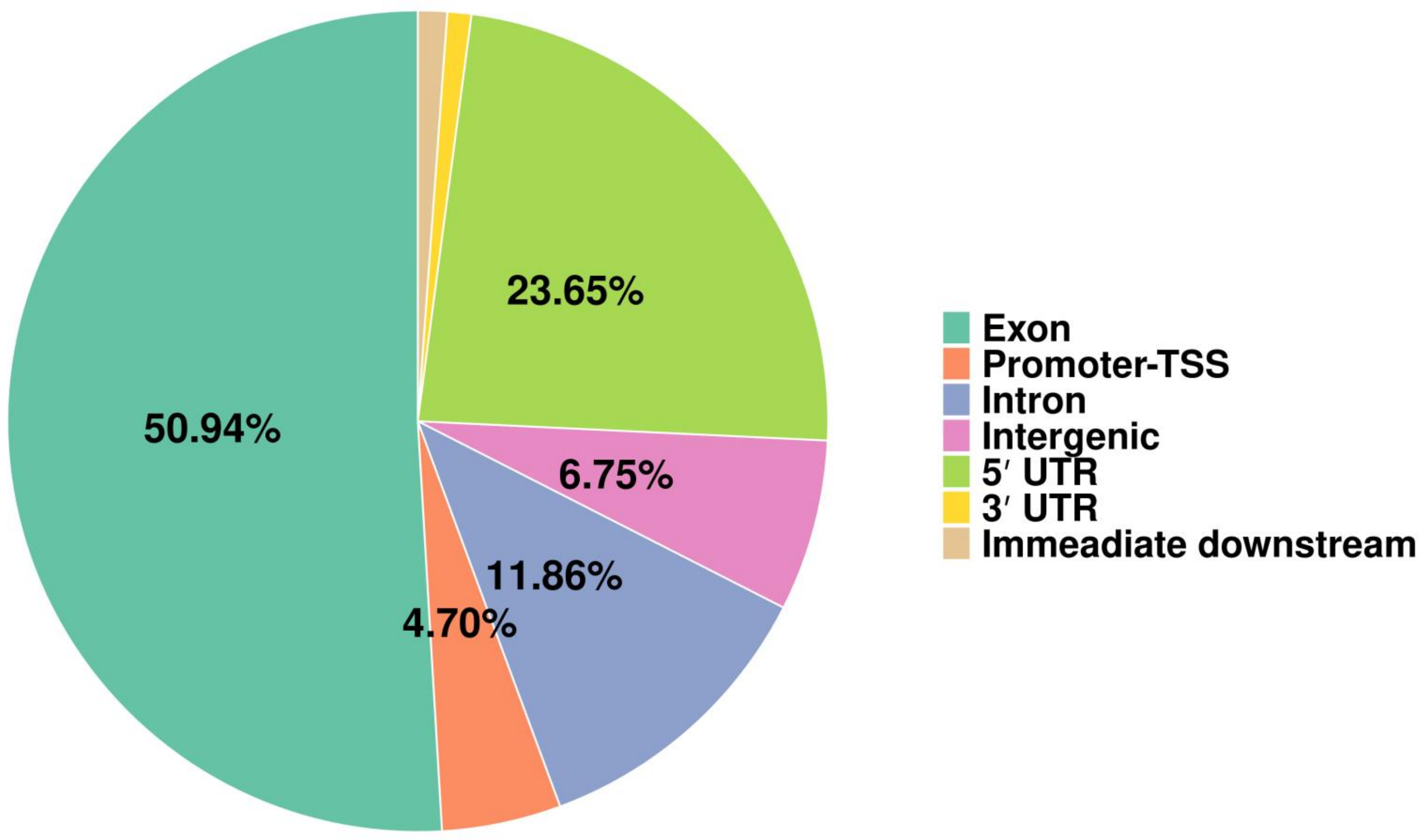

Supplementary Figure 9. UBP5 majorly targets protein coding regions. Pie-chart showing the distribution of annotated genic and intergenic regions in the UBP5 binding peaks.

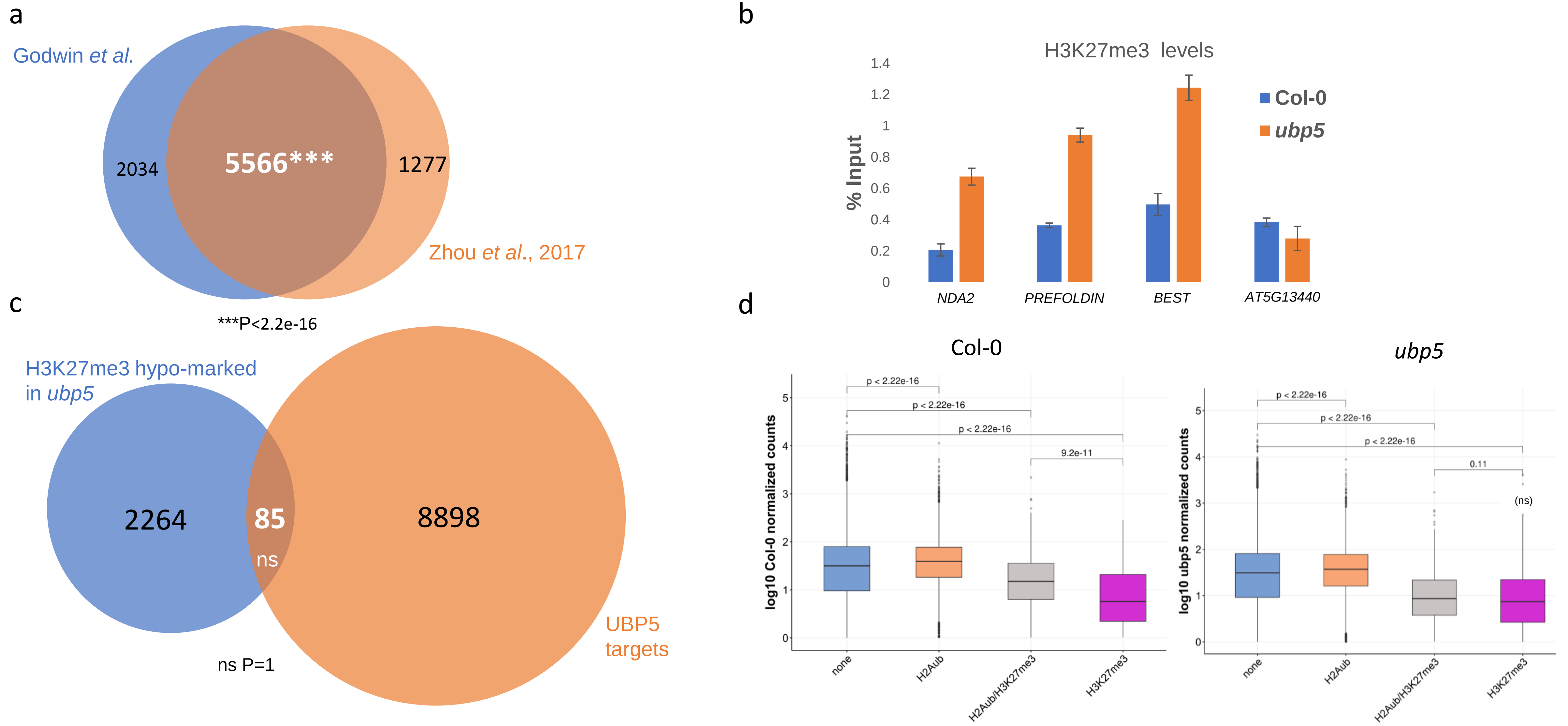

Supplementary Figure 10. H2K27me3 changes in *ubp5* influences transcription. a, Venn diagram representing the overlap of H3K27me3 marked genes in Col-0 seedlings between our dataset and Zhou et al., 2017. Super exact test was performed (*** represents significant overlap, $p<2.2e-16$). b, ChIP-qPCR validation of H3K27me3 gained genes in *ubp5*. Selected loci *ALTERNATIVE NAD(P)H DEHYDROGENASE 2 (NDA2* - AT2G29990), *PREFOLDIN* and *BEST* were among UBP5 targets transcriptionally downregulated in *ubp5*. AT5G13440 was selected as negative control. ChIP-qPCR results shown as % of input DNA. This data is a representation of one biological replicate. Error bars indicate SD of two technical replicates. c, Venn diagram representing H3K27me3 hypo-marked genes in *ubp5* and UBP5 targets, ns represents non-significant overlap. d, Box plots showing expression levels of only-H2Aub, both H2Aub/H3K27me3 and only-H3K27me3 marked genes of Col-0 (left graph) and *ubp5* (right graph). "None" represents those genes lacking H2Aub and H3K27me3 marks. The statistical significance of the differences was calculated using the Kruskal-Wallis test followed by Dunn's post hoc analysis, p-values are indicated, ns represents non-significant, N=4 biological replicates. For box and whiskers plot the middle line represents the median; the upper and lower lines are the first and third quartile (Q1 and Q3); the whiskers indicate the upper and lower limits of data spread by subtracting 1.5* interquartile range (IQR) from Q1 and adding 1.5* IQR to Q3.

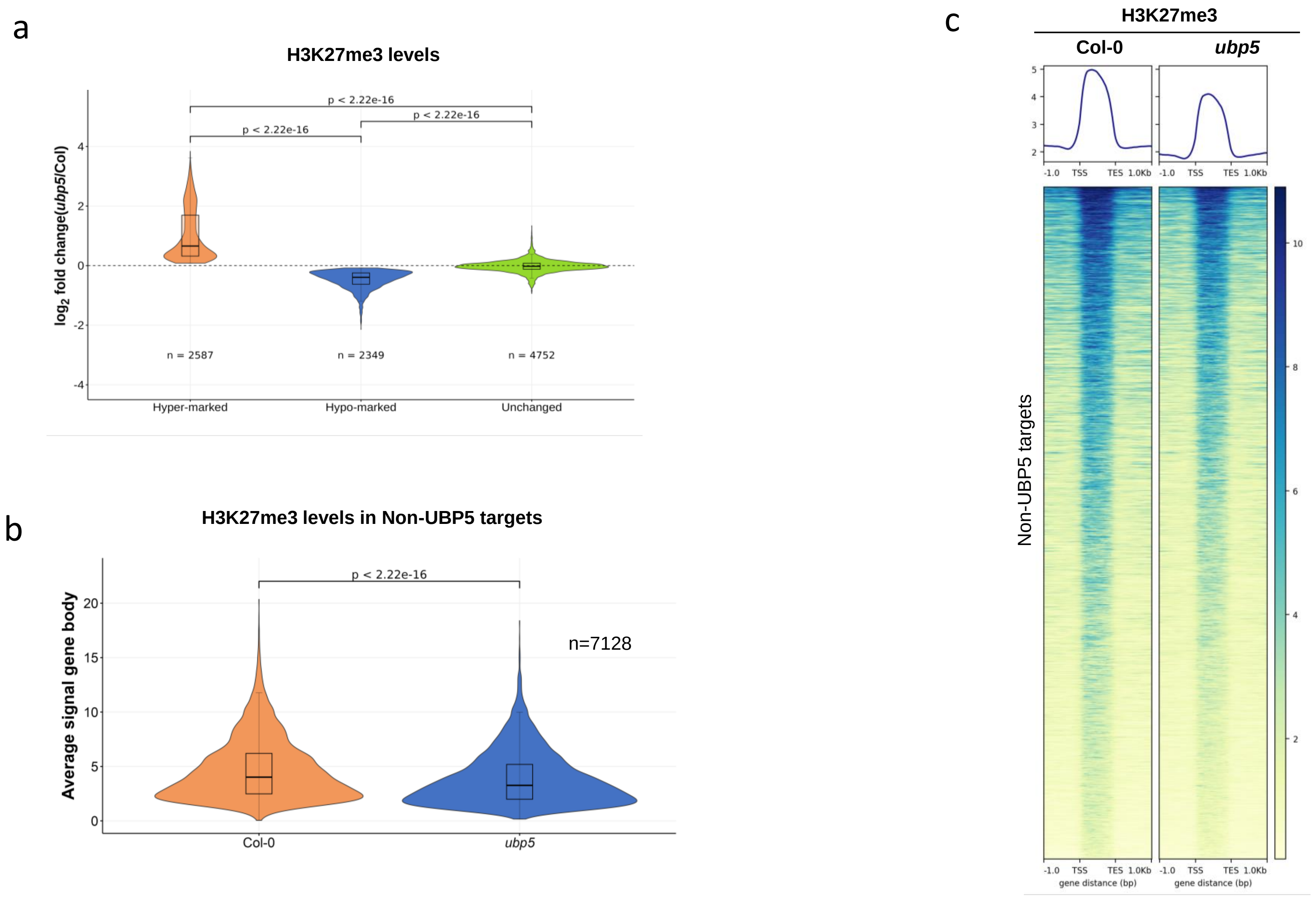

Supplementary Figure 11. H3K27me3 levels in different categories of genes. a, Average signal of H3K27me3 at gene body for hyper-marked, hypo-marked and unchanged. Statistical significance is tested using the Kruskal-Wallis test followed by Dunn's post hoc analysis, p values are indicated above the plot. b, H3K27me3 levels in the non-UBP5 targets. Violin cum box plots represents the average signal of H3K27me3 at gene body for non-UBP5 targets in Col-0 and *ubp5*. Statistical significance is tested according to Wilcoxon rank sum test, p-values are indicated above the plot. The violin plots show the distribution pattern of data and are overlaid with boxplots. For box and whiskers plot the middle line represents the median; the upper and lower lines are the first and third quartile (Q1 and Q3); the whiskers indicate the upper and lower limits of data spread by subtracting 1.5* interquartile range (IQR) from Q1 and adding 1.5* IQR to Q3. c, Heatmap showing the distribution of H3K27me3 on non-UBP5 targets (n=7,128) for Col-0 and *ubp5*.

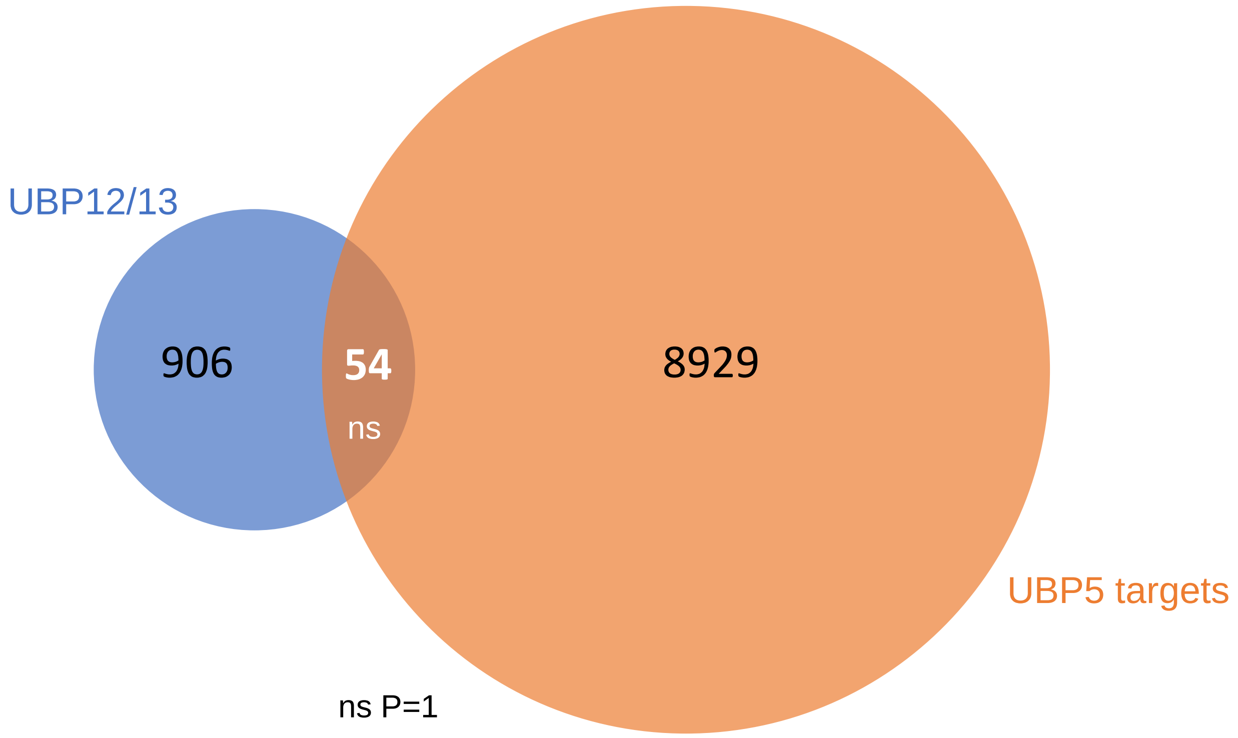

Supplementary Figure 12. UBP5 shows low overlap with UBP12/13. Venn diagram showing the overlap between UBP12/13 regulated genes and UBP5 target genes. UBP12/13 regulated genes are defined as protein-coding genes gaining H2Aub and not losing H2Aub in the first 1kb of the gene body in *ubp12/13* mutants. UBP12/13 data from Kralemann et al., 2020. Significance of overlap was tested using Super exact test, ns represents non-significance.

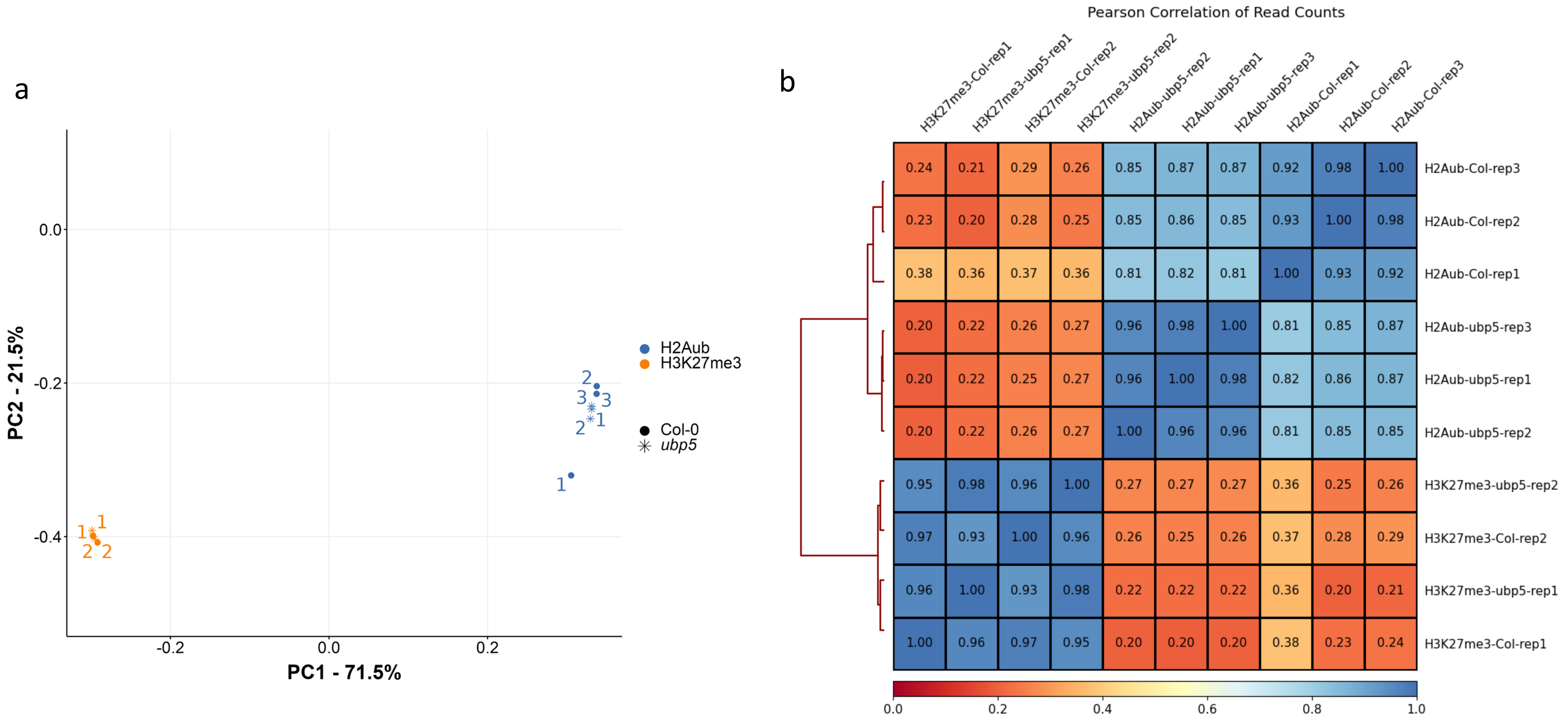

Supplementary Figure 13. Quality control of ChIP-seq replicates were done using PCA and correlation plots using Deeptools. a, Principal Component Analysis (PCA) plot showing the similarity/differences between the replicates of H2Aub and H3K27me3. b, Pairwise correlation matrix based on Pearson correlation coefficient for all possible combinations of H2Aub and H3K27me3 replicates in both Col-0 and *ubp5*. Both the analysis was done on binned and RPGC normalised bigwig files.

Primer name	Sequence
**UBP5 Genotyping_Forward primer**	TCTTTGGTATGTTGGGTCATGT
**UBP5 Genotyping_Reverse primer**	AGGCAAACTCTCCAGGCAAG
**UBP5_guide6_Bs_Forward primer**	ATATATGGTCTCGATTGTACGGGGGTGGTCCAACTCGTT
**UBP5_guide6-Forward primer**	TGTACGGGGGTGGTCCAACTCGTTTTAGAGCTAGAAATAGC
**UBP5_guide20-Reverse primer**	ACCAGTCGGAGGAGTTGCTTTTCAATCTCTTAGTCGACTCTAC
**UBP5_guide20-Bs_Reverse primer**	ATTATTGGTCTCGAAACAGTCGGAGGAGTTGCTTTTCAA

Supplementary Table 1: Primers designed for deletion mutagenesis of *UBP5* by the CRISPR/Cas9 system and primers for genotyping of the *ubp5* mutant.
