# Supplementary material for: The UBP5 histone H2A deubiquitinase counteracts PRCs-mediated repression to regulate Arabidopsis development
Source: Nat Commun. 2024 Jan 22;15:667. doi: 10.1038/s41467-023-44546-8 (PMC10803359; doi:10.1038/s41467-023-44546-8)
Supplement: Supplementary file 3 — Description of Additional Supplementary Files [file 41467_2023_44546_MOESM3_ESM.pdf]

Description of Additional Supplementary Files

Supplementary Data 1 RNA seq seedling_ubp5 and Col_0

Supplementary Data 2 Misregulated genes in ubp5_Developmental process

Supplementary Data 3 H2Aub and H3K27me3 marked genes in Col and ubp5 seedlings

Supplementary Data 4 Differential analysis of H2Aub marked genes in ubp5

Supplementary Data 5 Different categories of H2Aub marked genes

Supplementary Data 6 UBP5 target genes

Supplementary Data 7 Differential analysis of H3K27me3 marked genes in ubp5
