# Supplementary material for: The UBP5 histone H2A deubiquitinase counteracts PRCs-mediated repression to regulate Arabidopsis development
Source: Nat Commun. 2024 Jan 22;15:667. doi: 10.1038/s41467-023-44546-8 (PMC10803359; doi:10.1038/s41467-023-44546-8)
Supplement: Supplementary file 11 — Reporting Summary [file 41467_2023_44546_MOESM11_ESM.pdf]

nature portfolio

Corresponding author(s): Sara Farrona

Last updated by author(s): Dec 7, 2023



# Reporting Summary

Nature Portfolio wishes to improve the reproducibility of the work that we publish. This form provides structure for consistency and transparency in reporting. For further information on Nature Portfolio policies, see our Editorial Policies and the Editorial Policy Checklist.

## Statistics

For all statistical analyses, confirm that the following items are present in the figure legend, table legend, main text, or Methods section.

| n/a | Confirmed | |
|---|---|---|
| ☐ | ☒ | The exact sample size (*n*) for each experimental group/condition, given as a discrete number and unit of measurement |
| ☐ | ☒ | A statement on whether measurements were taken from distinct samples or whether the same sample was measured repeatedly |
| ☐ | ☒ | The statistical test(s) used AND whether they are one- or two-sided<br>*Only common tests should be described solely by name; describe more complex techniques in the Methods section.* |
| ☐ | ☒ | A description of all covariates tested |
| ☐ | ☒ | A description of any assumptions or corrections, such as tests of normality and adjustment for multiple comparisons |
| ☐ | ☒ | A full description of the statistical parameters including central tendency (e.g. means) or other basic estimates (e.g. regression coefficient) AND variation (e.g. standard deviation) or associated estimates of uncertainty (e.g. confidence intervals) |
| ☐ | ☒ | For null hypothesis testing, the test statistic (e.g. *F*, *t*, *r*) with confidence intervals, effect sizes, degrees of freedom and *P* value noted<br>*Give P values as exact values whenever suitable.* |
| ☒ | ☐ | For Bayesian analysis, information on the choice of priors and Markov chain Monte Carlo settings |
| ☒ | ☐ | For hierarchical and complex designs, identification of the appropriate level for tests and full reporting of outcomes |
| ☐ | ☒ | Estimates of effect sizes (e.g. Cohen's *d*, Pearson's *r*), indicating how they were calculated |

*Our web collection on statistics for biologists contains articles on many of the points above.*

## Software and code

Policy information about availability of computer code

| | |
|---|---|
| Data collection | No software is used for data collection |
| Data analysis | RNA-seq data analysis: Fastq file for read 55 nt R2 of pooled library was demultiplexed into separate fastq files for each replica using BRBseqTools (v 1.6). Demultiplex with parameters -p UB -UMI 14 -n 1. Read R2 of each library was trimmed to remove potential contamination with poly(A) tail using BRBseqTools (v 1.6) Trim and parameters -polyA 10 -minLength 30. Then mapped using STAR (v 2.7.8a) with parameters --sjdbOverhang 54 --outSAMtype BAM SortedByCoordinate --outFilterMultimapNmax 1. Finally, the deduplicated counts for each gene were obtained using BRBseqTools (v 1.6) CreateDGEMatrix with parameters -p UB -UMI 14 -s yes. Counts were used for differential gene expression analysis using DESeq2 R package.<br><br>The custom code used for the analysis has been deposited at [https://github.com/mohang13/ubp5_nat_comms] |

For manuscripts utilizing custom algorithms or software that are central to the research but not yet described in published literature, software must be made available to editors and reviewers. We strongly encourage code deposition in a community repository (e.g. GitHub). See the Nature Portfolio guidelines for submitting code & software for further information.

## Data

Policy information about availability of data

All manuscripts must include a data availability statement. This statement should provide the following information, where applicable:

- Accession codes, unique identifiers, or web links for publicly available datasets
- A description of any restrictions on data availability
- For clinical datasets or third party data, please ensure that the statement adheres to our policy

All data supporting the findings of this study are available within the manuscript and its supplementary files. All high-throughput sequencing data generated in this study have been deposited in GEO with accessions codes GSE217614 and GSE209707. Seeds of the ubp5 CRISPR/Cas9 line are available under request. Source data are provided with this paper.

## Research involving human participants, their data, or biological material

Policy information about studies with human participants or human data. See also policy information about sex, gender (identity/presentation), and sexual orientation and race, ethnicity and racism.

| | |
|---|---|
| Reporting on sex and gender | Not applicable |
| Reporting on race, ethnicity, or other socially relevant groupings | Not applicable |
| Population characteristics | Not applicable |
| Recruitment | Not applicable |
| Ethics oversight | Not applicable |

Note that full information on the approval of the study protocol must also be provided in the manuscript.

# Field-specific reporting

Please select the one below that is the best fit for your research. If you are not sure, read the appropriate sections before making your selection.

- [x] Life sciences
- [ ] Behavioural & social sciences
- [ ] Ecological, evolutionary & environmental sciences

For a reference copy of the document with all sections, see nature.com/documents/nr-reporting-summary-flat.pdf

# Life sciences study design

All studies must disclose on these points even when the disclosure is negative.

| | |
|---|---|
| Sample size | For Root and hypocotyl length analysis, we used 20 seedlings for each genotype.<br>For RNA-seq analysis, we used 4 biological replicates for each genotype.<br>For qRT-PCR, we used 3 biological replicates for each genotype.<br>For ChIP-qPCR analysis, we used 2 biological replicates for each genotype.<br>For WBs, we used 3 biological replicates.<br>For co-IP, we used 2 biological replicates.<br>For co-localisation analyses, we used 3 biological replicates.<br>For FRET-APB analyses, we used the following number of nuclei: PWO1-GFP N = 16; UBP5-GFP N = 15; UBP5-GFP+PWO1-mCh N = 7; UBP5-GFP+PWO1-mCh spec N = 14; PWO1-GFP+UBP5-mCh speckles N = 16; PWO1-GFP_mCh N = 15 |
| Data exclusions | We did not exclude any data. |
| Replication | Experimental findings were reliably reproduced as shown by error bars of independent replicates. All attempts at replication were successful. |
| Randomization | All samples were grouped based on the genotype, such as Col-0 (WT) and ubp5. Plants were placed randomly in the plant growth facility. |
| Blinding | In our study blinding was not relevant as it does not affect the behavior of Col-0 or ubp5 mutant plants as well as data analysis. |

# Reporting for specific materials, systems and methods

We require information from authors about some types of materials, experimental systems and methods used in many studies. Here, indicate whether each material, system or method listed is relevant to your study. If you are not sure if a list item applies to your research, read the appropriate section before selecting a response.

## Materials & experimental systems

| n/a | Involved in the study |
| --- | --- |
| ☐ | ☒ Antibodies |
| ☒ | ☐ Eukaryotic cell lines |
| ☒ | ☐ Palaeontology and archaeology |
| ☒ | ☐ Animals and other organisms |
| ☒ | ☐ Clinical data |
| ☒ | ☐ Dual use research of concern |
| ☐ | ☒ Plants |

## Methods

| n/a | Involved in the study |
| --- | --- |
| ☐ | ☒ ChIP-seq |
| ☐ | ☐ Flow cytometry |
| ☐ | ☐ MRI-based neuroimaging |

# Antibodies

Antibodies used

H2Aub antibody (Cell Signalling Technology D27C4, #8240, Dilution- 1:2000), anti-H2A antibody (Active Motif 91325, Dilution- 1:1000), anti-H3K27me3 antibody (Millipore 07-449, Lot no: 3253330, Dilution- 1:5000), anti-H3 (Abcam ab1791, Dilution- 1:5000), anti-GFP (Roche 11814460001, dilution-1:1000), anti-GFP antibody (Thermo Fisher A11122, 1:2000).

Validation

Ubiquityl-Histone H2A (Lys119) (Cell Signalling Technology D27C4): Rabbit monoclonal antibody is validated for:
W-Western IP-Immunoprecipitation IHC-Immunohistochemistry ChIP-Chromatin Immunoprecipitation

Anti-H3K27me3 antibody (Millipore 07-449): This rabbit polyclonal antibody has been independently validated for in WB and ChIP.

Anti-H2A antibody (Active Motif 91325): This rabbit polyclonal antibody is validated for western blot (WB) by Active Motif: WB: 2 µg/ml dilution.

Anti-H3 (Abcam ab1791): Rabbit polyclonal to Histone H3 - Suitable and validated for: ChIP, IP, WB.

Anti-GFP (Roche 11814460001): Monoclonal antibody for detection of both wild-type and mutant forms of GFP or GFP fusions using: IP, WB and Immunostaining.

Anti-GFP antibody (Thermo Fisher A11122): Polyclonal antibody is validated for WB, IHC, ChIP, IP.

# Plants

Seed stocks

Col-0-line seeds used in this study were obtained from Nottingham Arabidopsis Stock Centre (NASC). ubp5 CRISPR-Cas9 deletion mutant and UBP5prom::gUBP5-eGFP;ubp5 (complementation line) were generated in our lab.

Novel plant genotypes

The ubp5 deletion line was generated through Cas9-directed mutagenesis, employing floral dip transformation in A. thaliana. The transformation utilized a novel p3-Cas9-mCherry plasmid vector graciously supplied by the Charles Spillane lab, with comprehensive details on vector construction available in Dupouy et al., 2022. Design of the sgRNAs was facilitated by the CRISPR-P tool, and a double guide RNA was engineered to facilitate the deletion of a 3,361 bp fragment within the UBP5 gene. The primer designed for deletion mutagenesis is mentioned in the supplementary table1.

Authentication

We confirmed the genotype of the homozygous lines using PCR-based genotyping, including appropriate controls, with primer details provided in the supplementary table 1. Validation of the ubp5 CRISPR-Cas9 line was achieved by complementing this mutant with an UBP5prom::gUBP5-eGFP construct (a 1,708-kb-upstream fragment and gene-body regions of UBP5 without stop codon amplified from genomic DNA of Col-0 and fused to GFP by cloning the UBP5 genomic fragment within the pGKGWG vector). Our RT-qPCR analysis revealed no significant differences in UBP5 gene expression between the UBP5 complementation line and Col-0. This observation was further corroborated by phenotypic analysis, reinforcing the consistency of our findings.

# ChIP-seq

## Data deposition

☒ Confirm that both raw and final processed data have been deposited in a public database such as GEO.

☒ Confirm that you have deposited or provided access to graph files (e.g. BED files) for the called peaks.

Data access links
*May remain private before publication.*

https://www.ncbi.nlm.nih.gov/geo/query/acc.cgi?acc=GSE217614

Files in database submission

ChIP-seq raw data

hIP-seq raw data

H2Aub-Col-rep1
H2Aub-Col-rep2

H2Aub-Col-rep3
H2Aub-ubp5-rep1
H2Aub-ubp5-rep2
H2Aub-ubp5-rep3
H3K27me3-Col-rep1
H3K27me3-Col-rep2
H3K27me3-ubp5-rep1
H3K27me3-ubp5-rep2
Input-Col-rep1
Input-Col-rep2
Input-Col-rep3
Input-ubp5-rep1
Input-ubp5-rep2
Input-ubp5-rep3

UBP-GFP-rep1
UBP-GFP-rep2
Input

ChIP-seq processed data

H2Aub-Col-rep1.cov.RPGConly.bw
H2Aub-Col-rep2.cov.RPGConly.bw
H2Aub-Col-rep3.cov.RPGConly.bw
H2Aub-ubp5-rep1.cov.RPGConly.bw
H2Aub-ubp5-rep2.cov.RPGConly.bw
H2Aub-ubp5-rep3.cov.RPGConly.bw
H3K27-Col-rep1.cov.RPGConly.bw
H3K27-Col-rep2.cov.RPGConly.bw
H3K27-ubp5-rep1.cov.RPGConly.bw
H3K27-ubp5-rep2.cov.RPGConly.bw
Input-Col-rep1.cov.RPGConly.bw
Input-Col-rep2.cov.RPGConly.bw
Input-Col-rep3.cov.RPGConly.bw
Input-ubp5-rep1.cov.RPGConly.bw
Input-ubp5-rep2.cov.RPGConly.bw
Input-ubp5-rep3.cov.RPGConly.bw

H2Aub-Col-rep1_peaks.broadPeak
H2Aub-Col-rep2_peaks.broadPeak
H2Aub-Col-rep3_peaks.broadPeak
H2Aub-ubp5-rep1_peaks.broadPeak
H2Aub-ubp5-rep2_peaks.broadPeak
H2Aub-ubp5-rep3_peaks.broadPeak
H3K27-Col-rep1_peaks.broadPeak
H3K27-Col-rep2_peaks.broadPeak
H3K27-ubp5-rep1_peaks.broadPeak
H3K27-ubp5-rep2_peaks.broadPeak

UBP5-GFP-rep1.bw
UBP5-GFP-rep2.bw
Input-GFP.bw
UBP5_rep1_peaks.narrowPeak
UBP5_rep2_peaks.narrowPeak

Genome browser session
(e.g. UCSC)

Not applicable

## Methodology

Replicates

We used two biological replicates for H3K27me3, three biological replicates for H2Aub and two biological replicates for UBP5-GFP ChIP-seq.

Sequencing depth

All libraries are sequenced as paired-end.

| library | raw_reads | uniquely-mapped_reads |
|---|---|---|
| H2Aub-Col-rep1 | 64454814 | 17459312 |
| H2Aub-Col-rep2 | 87811678 | 19056892 |
| H2Aub-Col-rep3 | 72100112 | 32305726 |
| H2Aub-ubp5-rep1 | 63078438 | 20830962 |
| H2Aub-ubp5-rep2 | 60474880 | 7211785 |
| H2Aub-ubp5-rep3 | 89345678 | 52388274 |
| H3K27-Col-rep1 | 64735358 | 25300141 |
| H3K27-Col-rep2 | 85071198 | 25971019 |
| H3K27-Col-rep3 | 55360296 | 10719280 |
| H3K27-ubp5-rep1 | 65672826 | 28760690 |
| H3K27-ubp5-rep2 | 64395640 | 24363510 |
| H3K27-ubp5-rep3 | 45100326 | 17310561 |
| Input-Col-rep1 | 24645668 | 12790260 |
| Input-Col-rep2 | 68273690 | 39653714 |
| Input-Col-rep3 | 141803052 | 77976888 |
| Input-ubp5-rep1 | 24745166 | 13457398 |
| Input-ubp5-rep2 | 73033368 | 39718329 |
| Input-ubp5-rep3 | 105567682 | 51628738 |
| UBP5-GFP-Input | 62231578 | 57281205 |
| UBP5-GFP-rep1 | 97480944 | 82435999 |
| UBP5-GFP-rep2 | 855820454 | 738130222 |

Antibodies

H2Aub antibody (Cell Signalling Technology D27C4), anti-H3K27me3 antibody (Millipore 07-449), anti-GFP antibody (Thermo Fisher A11122).

Peak calling parameters

For UBP5-GFP ChIP

Read mapping:

```
bowtie2 -p 64 -x $REF_GENOME -1 $CHIP_FILE_1 -2 $CHIP_FILE_2 | samtools view -bS - > $ALIGNED_BAM
```

Peak calling:

```
macs3 callpeak -f BAMPE -B -c $ALIGNED_BAM_INPUT -t $ALIGNED_BAM_IP -g 110e6 -q 0.01 --outdir $OUT_DIR
```

For Histone marks ChIP-seq

Read mapping:

```
STAR --runThreadN 20 --genomeDir $REF_GENOME --outSAMtype BAM SortedByCoordinate --readFilesIn $CHIP_FILE_1
$CHIP_FILE_2 --alignIntronMax 1 --outFileNamePrefix $ALIGNED_BAM  --alignEndsType EndToEnd --readFilesCommand zcat --
outFilterMultimapNmax 1000 --outMultimapperOrder Random --outFilterMismatchNmax 2  --outFilterScoreMinOverLread 0 --
outFilterMatchNminOverLread 0
```

Peak calling:

```
macs3 callpeak -f BAMPE -B -c $ALIGNED_BAM_INPUT -t $ALIGNED_BAM_IP -g 110e6 --broad --broad-cutoff 0.1 --outdir $OUT_DIR
```

Data quality

Peaks were determined using parameters independently for each replicate. Only peaks present in two replicates were considered.

Software

The custom code used for the analysis has been deposited  and available public at [https://github.com/mohang13/ubp5_nat_comms]

# Flow Cytometry

## Plots

Confirm that:

- [ ] The axis labels state the marker and fluorochrome used (e.g. CD4-FITC).
- [ ] The axis scales are clearly visible. Include numbers along axes only for bottom left plot of group (a 'group' is an analysis of identical markers).
- [ ] All plots are contour plots with outliers or pseudocolor plots.
- [ ] A numerical value for number of cells or percentage (with statistics) is provided.

## Methodology

Sample preparation | *Describe the sample preparation, detailing the biological source of the cells and any tissue processing steps used.*

Instrument | *Identify the instrument used for data collection, specifying make and model number.*

Software | *Describe the software used to collect and analyze the flow cytometry data. For custom code that has been deposited into a community repository, provide accession details.*

Cell population abundance | *Describe the abundance of the relevant cell populations within post-sort fractions, providing details on the purity of the samples and how it was determined.*

Gating strategy | *Describe the gating strategy used for all relevant experiments, specifying the preliminary FSC/SSC gates of the starting cell population, indicating where boundaries between "positive" and "negative" staining cell populations are defined.*

☐ Tick this box to confirm that a figure exemplifying the gating strategy is provided in the Supplementary Information.

# Magnetic resonance imaging

## Experimental design

Design type | *Indicate task or resting state; event-related or block design.*

Design specifications | *Specify the number of blocks, trials or experimental units per session and/or subject, and specify the length of each trial or block (if trials are blocked) and interval between trials.*

Behavioral performance measures | *State number and/or type of variables recorded (e.g. correct button press, response time) and what statistics were used to establish that the subjects were performing the task as expected (e.g. mean, range, and/or standard deviation across subjects).*

## Acquisition

Imaging type(s) | *Specify: functional, structural, diffusion, perfusion.*

Field strength | *Specify in Tesla*

Sequence & imaging parameters | *Specify the pulse sequence type (gradient echo, spin echo, etc.), imaging type (EPI, spiral, etc.), field of view, matrix size, slice thickness, orientation and TE/TR/flip angle.*

Area of acquisition | *State whether a whole brain scan was used OR define the area of acquisition, describing how the region was determined.*

Diffusion MRI ☐ Used ☒ Not used

## Preprocessing

Preprocessing software | *Provide detail on software version and revision number and on specific parameters (model/functions, brain extraction, segmentation, smoothing kernel size, etc.).*

Normalization | *If data were normalized/standardized, describe the approach(es): specify linear or non-linear and define image types used for transformation OR indicate that data were not normalized and explain rationale for lack of normalization.*

Normalization template | *Describe the template used for normalization/transformation, specifying subject space or group standardized space (e.g. original Talairach, MNI305, ICBM152) OR indicate that the data were not normalized.*

Noise and artifact removal | *Describe your procedure(s) for artifact and structured noise removal, specifying motion parameters, tissue signals and physiological signals (heart rate, respiration).*

Volume censoring | *Define your software and/or method and criteria for volume censoring, and state the extent of such censoring.*

## Statistical modeling & inference

Model type and settings | *Specify type (mass univariate, multivariate, RSA, predictive, etc.) and describe essential details of the model at the first and second levels (e.g. fixed, random or mixed effects; drift or auto-correlation).*

Effect(s) tested | *Define precise effect in terms of the task or stimulus conditions instead of psychological concepts and indicate whether ANOVA or factorial designs were used.*

Specify type of analysis: ☐ Whole brain ☐ ROI-based ☐ Both

Statistic type for inference

(See Eklund et al. 2016)

*Specify voxel-wise or cluster-wise and report all relevant parameters for cluster-wise methods.*

Correction

*Describe the type of correction and how it is obtained for multiple comparisons (e.g. FWE, FDR, permutation or Monte Carlo).*

## Models & analysis

| n/a | Involved in the study |
|---|---|
| ☒ | ☐ Functional and/or effective connectivity |
| ☒ | ☐ Graph analysis |
| ☒ | ☐ Multivariate modeling or predictive analysis |
